# The SUMO conjugating enzyme UBC9 as a biomarker for cervical HPV infections

**DOI:** 10.3332/ecancer.2015.534

**Published:** 2015-04-29

**Authors:** Domenico Mattoscio, Chiara Casadio, Marzia Fumagalli, Mario Sideri, Susanna Chiocca

**Affiliations:** 1 European Institute of Oncology, Department of Experimental Oncology, Via Adamello 16, 20139 Milan, Italy; 2 European Institute of Oncology, Department of Pathology, 20141 Milan, Italy; 3 TTFactor Srl, Via Adamello 16, 20139 Milan, Italy; 4 European Institute of Oncology, Division of Gynecology, 20141 Milan, Italy; * Equally contributed to this work.

**Keywords:** Ubc9, SUMO, biomarker, HPV, LSIL, HSIL, cervical cancer

## Abstract

Human papillomaviruses (HPVs) infect stratified epithelium and are the causative agents of cervical cancer, the second most common cause of cancer-related death in women. A critical aspect that still persists in the HPV field is the selection of very sensitive and specific HPV diagnostic assays. Here, we provide evidence that the crucial small ubiquitin-like modifier (SUMO) E2-conjugating enzyme Ubc9 is strongly upregulated in cervical lesions. Ubc9 detection could thus be used in diagnosing and/or monitoring the progression of an HPV oncogenic infection.

## Background

Human papillomaviruses (HPVs) are a group of small, double-stranded DNA viruses known to be the primary cause of cervical cancer. Evidence is now emerging on their role in the aetiology of head and neck and other cancers as well in both women and men.

HPVs can be classified as low-risk and high-risk viruses. In the cervix, low-risk HPVs are associated with a spectrum of benign warts, whereas infections with high-risk HPVs, such as HPV16, are manifested by intraepithelial malignant progression caused by viral E6 and E7 [1, 2]. High-risk E6 and E7 are oncoviral proteins that, by respectively degrading the tumour suppressors p53 and pRb (retinoblastoma protein) through the ubiquitin–proteasome pathway, perturb cell cycle regulation and normal cellular functions in infected cells [1, 2].

Robust and convincing evidence now reinforces the acceptance of cervical cancer prevention approaches. The strategy is to strongly aim at and focus on persistent infection caused by HPV. Besides laboratory tests to detect HPV DNA, there are other promising markers of HPV oncogenic infection. These include the following: (i) markers of increased HPV mRNA and protein expression; (ii) markers of increased cell proliferation, such as Ki-67, MCM2, TOP2a, and p16INK4a; and (iii) markers of chromosomal instability, such as a gain of chromosome arm 3q and HPV DNA integration [3].

Currently, the most promising candidate as a clinical biomarker is detection of over-expressed p16INK4a (p16) [4–7]. p16 overexpression is a result of inactivation of the cell-cycle regulatory retinoblastoma protein (pRb) by high-risk HPV E7 [4–7]. p16 detection has now multiple clinical indications in both the screening and prevention programmes, either at cytology or at histopathology level [3].

The most used HPV diagnosis is still based on cytology, since the virus clearly alters the infected cells generating a characteristic vacuolisation around nuclei. This hallmark phenomenon is called koilocytosis and indicates the presence of an HPV infection. A koilocyte is a squamous epithelial cell that has undergone a number of structural changes which occur as a result of infection of the cell by HPV [8]. Such morphological changes are not necessarily dysplastic, as those present in cervical intraepithelial neoplasia (CIN). p16 has been recently proposed as a marker for cervical intraepithelial neoplasia 2 and 3 (CIN2 and CIN3) detection or as a triage test to identify HPV-positive women at risk of CIN3 development within 3 years. Thus, HPV p16-positive women would clearly benefit from prompt clinical intervention and colposcopy [5]. However, the p16 marker is not always effective in detecting CIN2 (Chiara Casadio and Mario Sideri personal communication).

Sumoylation is a post-translational modification where the small ubiquitin-like modifier (SUMO) proteins are reversibly attached to the protein target through an ubiquitin-like pathway, with many different outcomes on protein stability, interaction and localisation, DNA repair and replication, transcriptional regulation, cell cycle control, apoptosis, cell signalling, and viral replication [9–11]. Among their different ways to exploit host cellular systems, viruses are also known to target post-translational modification systems, such as the SUMO pathway (reviewed in [11–13]). In fact, most of the DNA viruses (parvoviruses, adenoviruses, papovaviruses, and herpesviruses) have viral proteins that are sumoylated or interact with SUMO components (reviewed in [11–13]). Conjugation of target proteins with SUMO requires a series of events catalysed by the E1 (SUMO-activating SAE1/SAE2), E2 (SUMO-conjugating Ubc9), and E3 (SUMO-ligating) enzymes family. Human tissues express four SUMO family members, SUMO1, the nearly identical SUMO2 and SUMO3, all ubiquitously expressed, and SUMO4, which is highly similar to SUMO2 but primarily expressed in kidney, lymph node, and spleen [9, 10, 14].

Our laboratory has previously demonstrated that other viral proteins, such as the adenoviral protein Gam1, can dysregulate the SUMO pathway by interacting with cellular proteins [14–20]. Recent data also described that high-risk HPV E6 and E7 oncoproteins are capable of targeting the sumoylation system through reducing the SUMO conjugating enzyme Ubc9 levels in cell lines [21] or by modulating sumoylation of host proteins [22, 23].

We thus asked whether we could assess differences in the expression of endogenous Ubc9 in HPV-positive cervical lesions from patients treated at the European Institute of Oncology (Milan, Italy).

The present studies show the upregulation of the sumoylation machinery conjugating enzyme Ubc9 in cervical biopsies in a lesion-dependent manner. Our data pinpoint to the possible use of Ubc9 as a biomarker for HPV oncogenic infection. Indeed, Ubc9 allows precise, sensitive, and selective detection of the infection.

## Methods

### Statistical analysis

One-way analysis of variance (ANOVA) followed by the Bonferroni test was performed using GraphPad Prism version 5.00 (GraphPad Software, La Jolla California USA, www.graphpad.com). Values of P < 0.05 were considered significant.

### Immunohistochemistry analysis

For immunohistochemistry, cervical samples were formalin-fixed and paraffin-embedded (FFPE) according to established procedures. All sections were counterstained with Mayer’s haematoxylin and visualised using a bright field microscope. Images were generated with a BX51 Upright Microscopes from Olympus America Inc. The following primary antibody was used: anti-Ubc9 (Santa Cruz, SC10759) (antigen retrieval at 99°C for 40 minutes in water bath, EDTA buffer pH 8.0, cool down to R.T. for 20 minutes), 1:3,200, incubation O/N at 4°C. The Envision Kit from DAKO was utilised, and after the chromogenic visualisation step using the 3,3′-diaminobenzidine (DAB) chromogen, slides were counterstained with haematoxylin and coverslipped.

## Results

### Immunohistochemistry analysis of cervical intraepithelial neoplasia

We assessed by immunohistochemistry (IHC) analysis whether there was a differential expression of Ubc9 in different grades of cervical intraepithelial neoplasia (CIN), also referred to as HSILs (high-grade squamous intraepithelial lesions) or LSILs (low-grade squamous intraepithelial lesions)) from HPV-positive patients. Typically, CIN1 refers to LSILs, whereas CIN2 and CIN3 to HSILs.

We analysed a total of approximately 130 patients, among which there were 54 LSILs and 78 HSILs (Table 1). In some patients, both low- and high-grade lesions were found, and thus, both lesions were scored. We also included adjacent normal tissues in the analysis.

**Figure 1.**
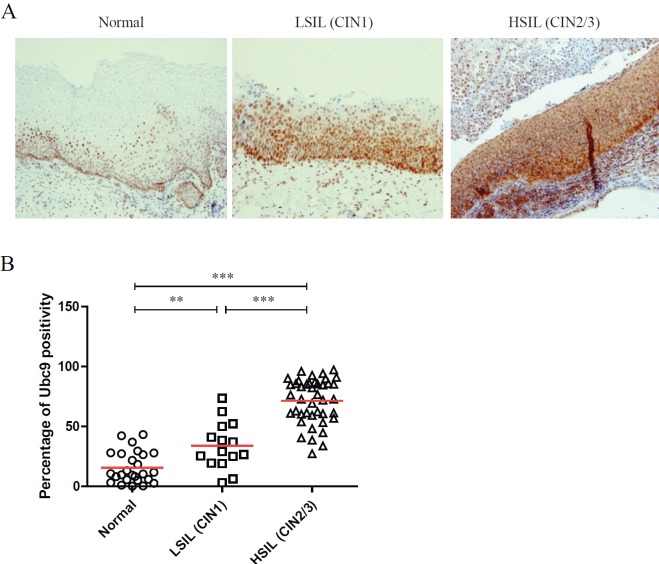
Ubc9 expression increases during cervical lesion progression. (A) Representative immunohistochemical (IHC) images of formalin-fixed and paraffin-embedded (FFPE) human cervical tissues. Normal, low-squamous intraepithelial lesions (LSIL-CIN1) and high-squamous intraepithelial lesion (HSIL-CIN2/3) were incubated with anti-Ubc9 antibody (Santa Cruz Biotechnology), counterstained with haematoxylin and visualised under a bright field microscope. (B) Percentage of Ubc9-positive cells in human cervical tissues. Ubc9-stained FFPE tissues were scored with Aperio Image-Scope (Leica Biosystems) using the positive pixel count algorithm. Data are expressed as percentage of positive plus strong positive stained pixels calculated in several slides obtained from at least 10 different donors. The red lines represent the mean for each group. Statistical significance was calculated with GraphPad Prism software using one-way ANOVA followed by Bonferroni post hoc test. **P < 0.001; ***P < 0.0001.

As demonstrated by the representative IHC reported in Figure 1A, we found that Ubc9 expression increases as lesions proceed from normal to a high grade. Indeed, in normal epithelium, Ubc9 is almost exclusively expressed in the basal cells, whereas in low-grade lesions, Ubc9 is also expressed in the midzone of the squamous epithelia. Finally, in HSILs, Ubc9 is expressed in high amounts almost in all the cells of the lesion.

Moreover, in some IHC experiments on FFPE cervical samples from the same cohort of patients, we also quantitated the percentage of Ubc9 positivity by computational analysis. As depicted in Figure 1B, as the cervical lesion progressed from LSIL to HSIL, there was a statistically significant increase in Ubc9 expression levels. Although non-infected tissues have a basal amount of Ubc9, the percentage of Ubc9 positivity strongly increases in low-grade cervical lesions. This phenotype is even more exacerbated in high-grade tissues (Figure 1B), again confirming that Ubc9 expression increases during cervical lesion progression. Thus, our data designate quantitative and qualitative Ubc9 expression as a potential excellent marker for cervical CIN1 and CIN2/3 lesion identification. Interestingly, Figure 1A shows that Ubc9 protein levels are also increased in the underlying stroma supporting the cancer lesion and again more so in HSILs. We are currently investigating this phenomenon.

## Discussion and conclusion

The concept of using Ubc9 and other components of the SUMO pathway as diagnostic markers has emerged from other studies as well.

Ronen *et al* suggested that Ubc9 may play a role in tumorigenesis and tumour progression in head and neck squamous cell carcinoma (HNSCC) and could potentially be used as a molecular marker for head and neck cancer progression [24].

Szendefi *et al* supported the use of promyelocyte protein-containing nuclear bodies and their association to SUMO-1, as a cytodiagnostic marker paralleling cervical cancer progression [25].

Wang *et al*. showed that expression of SENP1 (SUMO-specific protease 1, a member of the de-SUMOylation protease family) directly correlated with prostate cancer aggressiveness and recurrence [26]. Their results showed how SENP1 contributed to the progression of prostate cancer and suggest that SENP1 may be a prognostic marker and a therapeutic target for metastasis in patients with prostate cancer.

Chen *et al* evaluated the contribution of Ubc9 to chemoresistance in breast cancer patients. The expression level of Ubc9 was determined by IHC: the proportion of Ubc9-positive cells was higher in invasive ductal carcinoma compared to normal breast tissues. Furthermore, besides poor clinical response to chemotherapy, high Ubc9 expression associated with poor differentiation, larger tumour size, advanced clinical stage, lymph node metastasis, basal-like phenotype and thus overall worse clinical prognosis [27].

**Figure 2.**
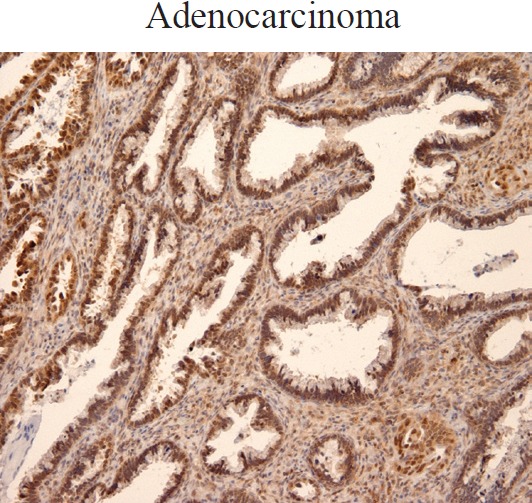
Ubc9 expression in cervical adenocarcinoma. Cervical adenocarcinoma FFPE was incubated with anti-Ubc9 antibody (Santa Cruz Biotechnology), counterstained with haematoxylin and visualised under a bright field microscope.

In the cohort of patients analysed, we also found increased Ubc9 expression in adenocarcinoma (Figure 2). Taken together, our results are consistent with studies reporting Ubc9 overexpression and/or contribution to tumorigenesis in multiple cancer types [24, 28–39, 40].

The precise diagnosis of LSILs and HSILs is the most important and key determinant to cervical cancer prevention. Today’s strategy implies that both the identification and the eradication of CIN2/3 help to prevent invasive cancer and to monitor LSILs towards clearance or possible progression to HSILs. The trigger to treatment is the histopathological diagnosis of CIN2/3, in which the present data are indicated as very sensitive to staining with an antibody that recognises the sumoylation pathway conjugating enzyme Ubc9. Furthermore, Ubc9 can also detect CIN1, thus making it a valuable marker to avoid overdiagnosis and therefore overtreatment.

Cervical cancer prevention is a worldwide problem, especially increasing in developing countries. To increase the screening power, pathologists must be trained to spot CIN2/3 diagnosis, with no doubt. Thus, Ubc9 staining could be very helpful, and there is clearly a market potential to implement this idea. In conclusion, proteins involved in the sumoylation pathway, in particular Ubc9, could be exploited towards cervical cancer prevention.

## Conflict of interest

The authors declare that they have no conflict of interest.

## Figures and Tables

**Table 1.** Patients samples evaluated for Ubc9 expression in IHC.

Characteristics	
**Age of Patients (years)** Mean Range	38 22–76
**Histopathological Diagnosis (number of specimens)** Negative LSIL (CIN1) HSIL (CIN2/3) Metaplasia Adenocarcinoma Squamous cell carcinoma NE	4 54 78 3 3 4 3

NE: not evaluated
